# *Asparagus cochinchinensis* alleviates disturbances of lipid metabolism and gut microbiota in high-fat diet-induced obesity mice

**DOI:** 10.3389/fphar.2022.1015005

**Published:** 2022-10-12

**Authors:** Shiyue Luo, Lixiao Zhou, Xuejun Jiang, Yinyin Xia, Lishuang Huang, Run Ling, Shixin Tang, Zhen Zou, Chengzhi Chen, Jingfu Qiu

**Affiliations:** ^1^ Department of Health Laboratory Technology, School of Public Health, Chongqing Medical University, Chongqing, China; ^2^ Center of Experimental Teaching for Public Health, Experimental Teaching and Management Center, Chongqing Medical University, Chongqing, China; ^3^ Research Center for Environment and Human Health, School of Public Health, Chongqing Medical University, Chongqing, China; ^4^ Department of Occupational and Environmental Health, School of Public Health, Chongqing Medical University, Chongqing, China; ^5^ Molecular Biology Laboratory of Respiratory Diseases, Institute of Life Sciences, Chongqing Medical University, Chongqing, China

**Keywords:** *Asparagus cochinchinensis*, obesity, high-fat diet, lipid metabolism, gut microbiota

## Abstract

*Asparagus cochinchinensis* is a valuable traditional Chinese medicine that has anti-inflammatory ability and effectively regulates the dysbiosis within the body. Obesity is usually characterized by chronic low-grade inflammation with aberrant gut microbiota. However, the role of *Asparagus cochinchinensis* against obesity remains unknown. Therefore, a high-fat diet (HFD)-induced obese mouse model with or without aqueous extract from *Asparagus cochinchinensis* root (ACE) treatment was established herein to determine whether ACE alleviated obesity and its involved mechanisms. Our results showed that ACE administration significantly decreased the weight gain and relieved dyslipidemia induced by HFD Treatment of ACE also improved glucose tolerance and insulin resistance in obese animal model, and remarkably decreased inflammation and lipogenesis in the liver and adipose. Moreover, administration of ACE significantly reshaped the gut microbiota of obese mice. These findings together suggest that ACE has beneficial effect against HFD-induced obesity and will provide valuable insights for the therapeutic potential of ACE against obesity and may aid in strategy-making for weight loss.

## Introduction

Obesity is a chronic metabolic disease with excessive accumulation and abnormal distribution of fat, and it promotes the occurrence or development of numerous disorders, such as cardiovascular diseases, diabetes, cancers, and osteoarthritis (Romieu et al., 2017; Piche et al., 2020; Rinonapoli et al., 2021). Since 1980, the prevalence of overweight is rapidly increasing, and about one-third of global population is determined to be obese or overweight (Ataey et al., 2020). Obesity and its complications have become one of the major public health problems, which place a tremendous burden on the health care systems. Although there are several available Food and Drug Administration (FDA)-approved anti-obesity medications, they remain hard to produce effective weight loss without inducing side effects (Heymsfield and Wadden, 2017). Therefore, it is an urgent need to find new treatment strategies for maintaining a healthy weight.

The fundamental cause of obesity or overweight is that the stored energy is more than the energy consumed by the body. After excessive nutrients intake or high-fat diet (HFD), excess energy will be stored in adipose cells. Then, pathological changes will be observed in the adipose tissues, such as accumulation of triglycerides, adipocyte hypertrophy and pro-inflammatory cytokines releasing (Lee and Shin, 2017). It is worth noting that pro-inflammatory cytokines not only induce systemic inflammation but also promote insulin resistance (Terzo et al., 2018; Nimptsch et al., 2019). The excessive accumulation of triglycerides in hepatocytes may result in hepatic steatosis or even more serious condition such as nonalcoholic steatohepatitis (Suarez et al., 2017).

Restriction of energy intake and improvement of lipid metabolism are main targets for obesity prevention and management. Accumulating studies have evidenced that gut microbiota plays a vital role in host nutrient absorption, energy consumption and lipid metabolism (Kindt et al., 2018; Aron-Wisnewsky et al., 2021). For instance, Liu et al., observed HFD can alter the gut microbial composition and subsequently changing the production of gut microbial metabolites (Liu et al., 2020). After feeding with a high-fat, sugar-rich diet, germ-free (GF) mice are protected from diet-induced obesity (DIO) when compared with control animals (Ng et al., 2014). Therefore, it is a promising strategy to control weight through modulating gut microbiota.

*Asparagus cochinchinensis* (Lour.) Merr. belongs to the genus *Asparagus* (Liliaceae). It is widely distributed in the temperate zone (China, Korea, Japan) to tropical Asia (Laos and Vietnam) (Lee et al., 2019). Steroidal saponins and polysaccharides are conceived as the major chemical components of *Asparagus cochinchinensis*, and the content of polysaccharides was higher in roots than in stems (Xue et al., 2022). Therefore, the *Asparagus cochinchinensis* root have been historically used in Chinese folk medicine for the treatment of cough, acute and chronic bronchitis, pharyngitis, hemorrhoids, and cancers for thousands of years (Jian et al., 2013). According to previous reports, the aqueous extract from *Asparagus cochinchinensis* root (ACE) possesses many biological functions including anti-inflammatory and anti-oxidation and anti-tumor effects (Lee et al., 2016; Lei et al., 2017; Lee et al., 2018). For example, ACE exerted significant anti-oxidant effects in AD mice, while intragastric administration of the *Asparagus cochinchinensis* root extracts can reduce phthalic anhydride (PA)-induced skin inflammation and loperamide-induced gastrointestinal inflammation in animals (Sung et al., 2016; Lee et al., 2018; Kim et al., 2019). In addition, an *in vitro* study revealed the ultrasonic extract of *Asparagus cochinchinensis* root had obvious effect against pathogenic bacteria including *Staphylococcus aureus*, *Escherichia coli* and *Aspergillus Niger* (Fang et al., 2012). Oxidative stress and inflammation link the fat accumulation-derived alterations and the development of multiple health problems. However, whether ACE can alleviate HFD-induced obesity remains unclear.

Therefore, this study aimed to investigate the effect of ACE on HFD-induced obesity and the potential mechanisms involved. Body weight and blood lipid contents were evaluated in the HFD-fed mice with or without ACE administration. Furthermore, the parameters related to lipid metabolism, inflammatory response as well as alterations of gut microbiota were assessed for revealing the involved mechanisms. These findings will provide novel evidence for therapeutic potential of ACE in preventing and treating obesity.

## Materials and methods

### Chemical and reagents

The high-fat diet containing 60% calories from fat (D12492) was purchased from Xietong Pharmaceutical Bio-engineering Co., Ltd. (Jiangsu, China). *Asparagus cochinchinensis* roots were purchased from Kangdi Medicine Industry Co., Ltd. (Chongqing, China, Lot: 211101) and were authenticated corresponding authors. Glucose solution was purchased from Tisansheng Pharmaceutical Co., Ltd. (Hubei, China). Insulin was purchased from Novo Nordisk Pharmaceutical Co., Ltd. (Tianjin, China). Commercial kits for total cholesterol (TC), triglyceride (TG), high-density lipoprotein (HDL), low-density lipoprotein (LDL), alanine aminotransferase (ALT), and aspartate aminotransferase (AST) were obtained from Nanjing Jiancheng Bioengineering Institute (Nanjing, Jiangsu, China). Hematoxylin and eosin (H&E) staining kit and Oil Red O staining kit were both purchased from Solarbio Science and Technology Co., Ltd. (Beijing, China). Cluster of differentiation 68 (CD68) and adhesion G protein-coupled receptor E1 (F4/80) antibodies were purchased from Protetintech Group, Inc. (Rosemont, United States).

### Pharmaceutical preparation

The pharmaceutical preparation was performed according to procedures reported previously (Fang et al., 2012), Briefly, extraction was done using a maceration method with a 1:10 ratio of the material and distilled water. In detail, about 100 g of *Asparagus cochinchinensis* dry roots was weighted and immersed in cold water for 20–30 min. Then, the decoction of *Asparagus cochinchinensis* roots was prepared by decoction twice by adding water, 600 mL for the first time and 400 mL for the second time. For each time, the heating was continued with a small fire for 30 min after boiling. Thereafter, the obtained solution from decoction was cooled down and filtered through four layers of gauze to remove the residue. The filtered solution was consolidated and dried with a vacuum Rotary evaporator (HUXI, RE-85C, Shanghai, China) and the yield of ACE is 44.78 g (extraction rate 44.78%). Then, the extracts were dissolved in distilled water to a stock solution of 0.45 g/mL. After high-pressure sterilization, the ACE solution was stored at 4°C for subsequent analysis. The stored solution was kept free from any contamination. Before intragastric administration, ACE solution were further diluted to the required concentration with distilled water.

### Liquid chromatography–mass spectrometry (LC–MS)

LC-MS was used to identify the monomeric compounds in the ACE and the protocol was carried out according to previous report (Gao et al., 2022). In brief, 500 μL of ACE was diluted by adding 1 mL methanol and filtered through a 0.22 μm filter. Thereafter, 10 μL of the diluted sample was analyzed by using a Hybrid Quadrupole-TOF LC/MS/MS Mass Spectrometer (Component ID: Triple TOF5600+, Manufacturer. AB Sciex Instruments; U300). The chromatographic conditions were set as follows: Wates BEH C18 (150 × 2.1 mm, 1.7 µm) was used as chromatographic column, the column temperature was 40°C, the total flow rate was set at 0.3 mL/min. The mobile phases were A. 0.1% HCOOH-H_2_O and B. acetonitrile and the mobile phase gradient was shown in supplementary data Supplementary Table S1. Mass spectrometric analysis was performed in electrospray ionization (ESI) positive and negative ion modes. The secondary mass spectra were obtained from information dependent acquisition (IDA) in high sensitivity mode, Declustering potential (DP): ±60 V, Collision Energy: 35 ± 15 eV. The original data were imported into MS-Dial 4.70 software, and the peak information was searched through three databases MassBank, Respect, and GNPS (14,951 records in total). The analysis results were shown in supplementary data Supplementary Table S2.

### Animal models and ACE administration

A total of 36 healthy male C57BL/6J mice at 4-weeks-old (12–16 g) were purchased from the Experimental Animal Center of Chongqing Medical University (Chongqing, China, license numbers: SCXK(Yu)2018–003). Mice were kept in the Experimental Animal Center of Chongqing Medical University under constant conditions. The room temperature was set at (23 ± 1) °C and the humidity was set at (50 ± 10) %. Mice were all maintained in a standard 12 h:12 h light-dark cycle. After 1 week of acclimation, animals were randomly divided into the following four groups by using a randomization tool on the website https://www.randomizer.org: 1) Normal chow diet group (ND), 2) High-fat (60% energy from fat, 20% energy from protein) diet group (HFD), 3) ND with intragastric administration of ACE (450 mg/kg BW) group (ND + ACE) and 4) HFD with intragastric administration of ACE (450 mg/kg BW) (HFD + ACE) group. Each group had nine mice. The concentration of ACE was selected primarily based on the following two reasons. First, in the standard crude drugs of Chinese Pharmacopoeia, the dosage of *Asparagus cochinchinensis* root was recommended at 6–12 g for each adult. Herein, the administration dosage of *Asparagus cochinchinensis* root crude drug was calculated with the following formula based on the consideration of uncertain factor between mouse and human: crude drug dosage per kilogram of mice = adult crude drug dosage (g)/adult body weight × 12.3 (Nair and Jacob, 2016). Thus, the concentration of ACE for mouse should be 1.23–2.46 g/kg (crude drug). Second, the dose used in this study was also chosen according to the previous studies. For instance, mice were orally administrated with ACE at 400 mg/kg, 500 mg/kg, 600 mg/kg and 2000 mg/kg doses in previously published reports (Choi et al., 2018; Ou et al., 2013; Sung et al., 2017). Therefore, in this study, a similar ACE dose of 450 mg/kg once a day was applied to mice in this study. During the treatment, all the mice were free access to rodent chow and tap water, and their body weight was measured and recorded every 3 days. All the animals in this study were treated according to the National Institutes of Health Guide for the Care. The animal protocols of this study were reviewed and approved by the Animal Administration and Ethics Committee of Chongqing Medical University.

### Animal sacrifice and tissue collection

After designed treatment, all the mice were fasted for 6 h and sacrificed after anesthetizing with ketamine (80 mg/kg) and xylazine (6 mg/kg). The liver tissue, perirenal white adipose tissue (Per-WAT), epididymal white adipose tissue (Epi-WAT), and blood of each mouse were collected for subsequent analyses. The fat index was calculated as follows:

$$Fat\;index=\left[\begin{array}{c} \frac{Per\_WAT+Epi\_WAT}{Body}\;\\ weight \end{array}\right]\times 100\%$$

### Glucose tolerance and insulin resistance tests

Intraperitoneal (IP) glucose tolerance test (IPGTT) was carried out according to the protocols described in previous report (Tian et al., 2020). In short, overnight fasted mice were intraperitoneally injected with glucose solution (2 g/kg of body weight) at the 8^th^ week. The blood glucose (BG) levels at 0, 30, 60, and 120 min were measured by tail bleeding using a glucometer (Sinocare, Changsha, China). The area under the curve (AUC) was calculated using the following equation.

$$AUC=0.5\times \frac{BG0+BG30}{2}+0.5\times \frac{BG30+BG60}{2}+1\times \frac{BG60+BG120}{2}$$

For IP insulin tolerance test (IPITT), at 9^th^ week, the mice were injected intraperitoneally with 0.5 U/kg insulin after 4 h of fasting (Rial et al., 2020). The blood glucose concentration was measured at 0, 30, 60 and 120 min by using a glucose meter. Similar to the calculation for glucose tolerance, the area under the curve (AUC) was used to reflect insulin tolerance.

### Biochemical analysis

The blood was obtained by using the enucleation method. The collected blood was centrifuged at 3,000 × *g* for 15 min to obtain plasma. The levels of TC, TG, HDL, LDL, ALT, and AST in plasma were determined according to the manufacturer’s instructions of the commercial assay kits.

### Histology analysis of the liver and adipose tissues

The collected liver samples were fixed in freshly prepared 4% paraformaldehyde, while Epi-WAT tissues were fixed with polymethyl calcium. Each tissue was used for the preparation of at least five slides. After that, histology analysis of the liver and adipose tissues was performed by Hematoxylin-eosin (H&E) staining and Oil red O staining according to protocols described previously (Lu et al., 2020). For H&E staining, the paraffin slices were baked in a 65°C oven for at least 1.5 h to ensure adequate melting of the paraffin wax. Then, the sections were placed in xylene twice to remove the melted paraffin and dehydrated in gradient ethanol. Sections were sequentially stained with hematoxylin and eosin, and then dehydrated with ethanol. After that, sections were cleared twice in xylene and fixed with neutral resin.

For Oil red O staining, frozen sections were fixed in 10% formalin for 10 min, stained with Oil red O for 10 min, and washed with 75% ethanol followed by counterstaining with hematoxylin. After staining, the sections were immersed for 2 min in distilled water and fixed with glycerol. Finally, sections were observed using an optical microscope (Olympus, IX53, Tokyo, Japan).

### Immunohistochemistry assay

Immunohistochemistry assay was performed according to the protocols reported previously (Diao et al., 2021). In brief, the paraffin sections were deparaffinized in xylene and subjected to conventional gradient ethanol dehydration. Subsequently, the sections were quenched with fresh 3% hydrogen peroxide for 10 min to inhibit endogenous tissue peroxidase activity. After washing with phosphate buffer solution, the sections were incubated with a blocking buffer at room temperature for 30 min, followed by incubation with anti-lysozyme primary antibody at 4°C overnight. Subsequently, the sections were rewarmed at room temperature for 15 min, washed with phosphate buffer solution, and incubated with biotinylated secondary antibody for 1 h. Then, sections were incubated with horseradish enzyme-labeled streptavidin for 10 min. Finally, the sections were stained with 3, 3′-diaminobenzidine, counterstained with hematoxylin, mounted on coverslips and observed under a light microscope (Olympus, IX53, Tokyo, Japan). The images were evaluated using ImageJ software (NIH, Bethesda, MD, United States).

### Real-time quantitative PCR (RT-qPCR)

The RT-PCR was performed based on the protocols reported previously (Xiao et al., 2021). In brief, total RNA was extracted using RNeasy mini kit (Promega). PrimeScript™ RT Master Mix (Perfect Real Time, obtained from Takara Co., Ltd., Tokyo, Japan) was used to synthesize cDNA from 1 μg of total RNA. Quantitative PCR was performed with the TB Green™ Premix Ex Taq™ II (Tli RNaseH Plus) on CFX Connect™ Real-Time System (Bio-Rad). All primers used in this work were synthesized by Sangon Biotechnology Co., Ltd. (Shanghai, China) and the sequences were listed in Supplementary data Supplementary Table S3. The mRNA relative abundance was calculated and normalized to the mean expression of β-actin and the expression level of each gene was calculated by the 2^−ΔΔCt^ method.

### 16S ribosomal RNA gene sequencing

The 16S ribosomal RNA (rRNA) gene sequencing was performed according to the protocols described previously (Diao et al., 2021). At the end of treatment, fresh fecal of each mouse was collected under sterile conditions and stored at −80°C for microbiota analysis. The integrity of total genomic DNA was verified by 1% agarose gel electrophoresis. Then, the V3 and V4 regions of bacterial 16S rRNA gene were amplified in an ABI GeneAmp^®^9,700 polymerase chain reaction (PCR) system (Thermal cyclers from Applied Biosystems, CA, United States) using two universal primers 338F (5′-ACT​CCT​ACG​GGA​GGC​AGC​AG-3′) and 806R (5′-GGACTACHVGGGTWTCTAAT-3′). The PCR products were excised from agarose and purified by AxyPrep DNA Gel Extraction Kit (Axygen Biosciences, CA, United States). Subsequently, the PCR products were quantified by QuantiFluor™-ST Blue Fluorescence Quantification System (Promega Co., WI, United States). The MiSeq library was constructed for the preparation of the fragment DNA by TruSeq™ DNA Sample Prep Kit. The raw sequence reads were obtained by Illumina MiSeq platform at Majorbio Bio Tech Co. Ltd. (Shanghai, China).

### 16S ribosomal RNA gene sequencing analysis

Based on Usearch software program (version 7.1), the raw sequence readings were clustered into operational taxonomic units (OTUs) with 97% similarity using Majorbio’s cloud website located at https://cloud.majorbio.com (Majorbio, Shanghai, China). Observed species and alpha diversity indices, Sobs, Shannon, Simpson, Chao, and Ace were calculated using Mothur software programs (version v.1.30.1). β-diversity was measured by principal coordinate analysis (PCoA) and partial least squares discriminant analysis (PLS-DA). The linear discriminant analysis (LDA) coupled with effect size analysis (LEfSe) was used to identify the differential bacterial taxa within different groups (*p* < 0.05 and LDA score of ≥2.0).

### Statistical analysis

All the data were presented as the mean ± standard error of the mean (S.E.M). The statistical analysis was performed using independent student-*t*-test or two-way analysis of variance. Graphs were made by using GraphPad Prism 8.0 (GraphPad Software, La Jolla, CA). A *p* < 0.05 was considered as statistically significant.

## Results

### ACE reduced high-fat diet-induced body weight gain and fat deposition

**FIGURE 1:**
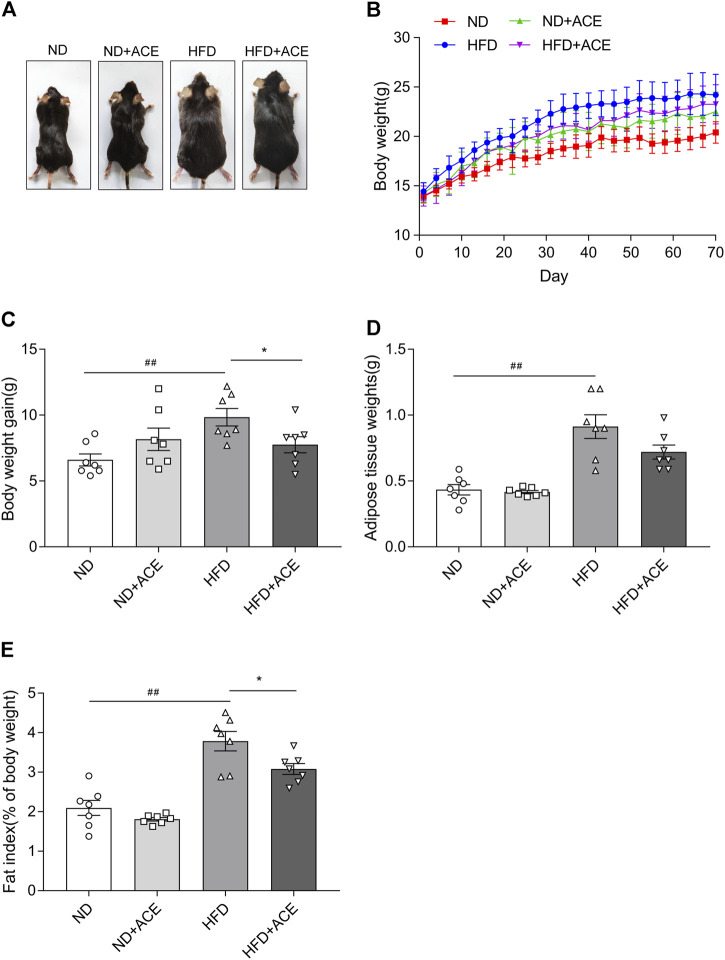
ACE reduced HFD-induced body weight gain and fat deposition. **(A)** Representative pictures of mice. **(B)** Body weight gain of mice during treatment. **(C)** Total weight gain at the end of treatment. **(D)** The weight of white adipose tissues. **(E)** Fat index. Data were shown as means ± S.E.M (n = 7 per group). Statistical analysis was conducted by using an independent student-*t* test. ^##^denotes *p* < 0.01 when compared with the ND group; *denotes *p* < 0.05 when compared with the HFD group.

The body weight and fat deposition of mice were shown in Figure 1. Compared with the ND group, the body weight, fat tissue weight, and fat index were all significantly increased in mice from the HFD group. In contrast, ACE administration remarkably reduced weight gain caused by the HFD (*p* < 0.05; Figures 1A–C). There were no significant differences in the fat tissue weight, fat index decreased significantly after ACE administration (*p* < 0.05; Figures 1D,E). These results indicate that ACE can suppress body weight gain and fat deposition induced by HFD.

### ACE ameliorated high-fat diet-induced abnormal blood metabolic parameters

**FIGURE 2:**
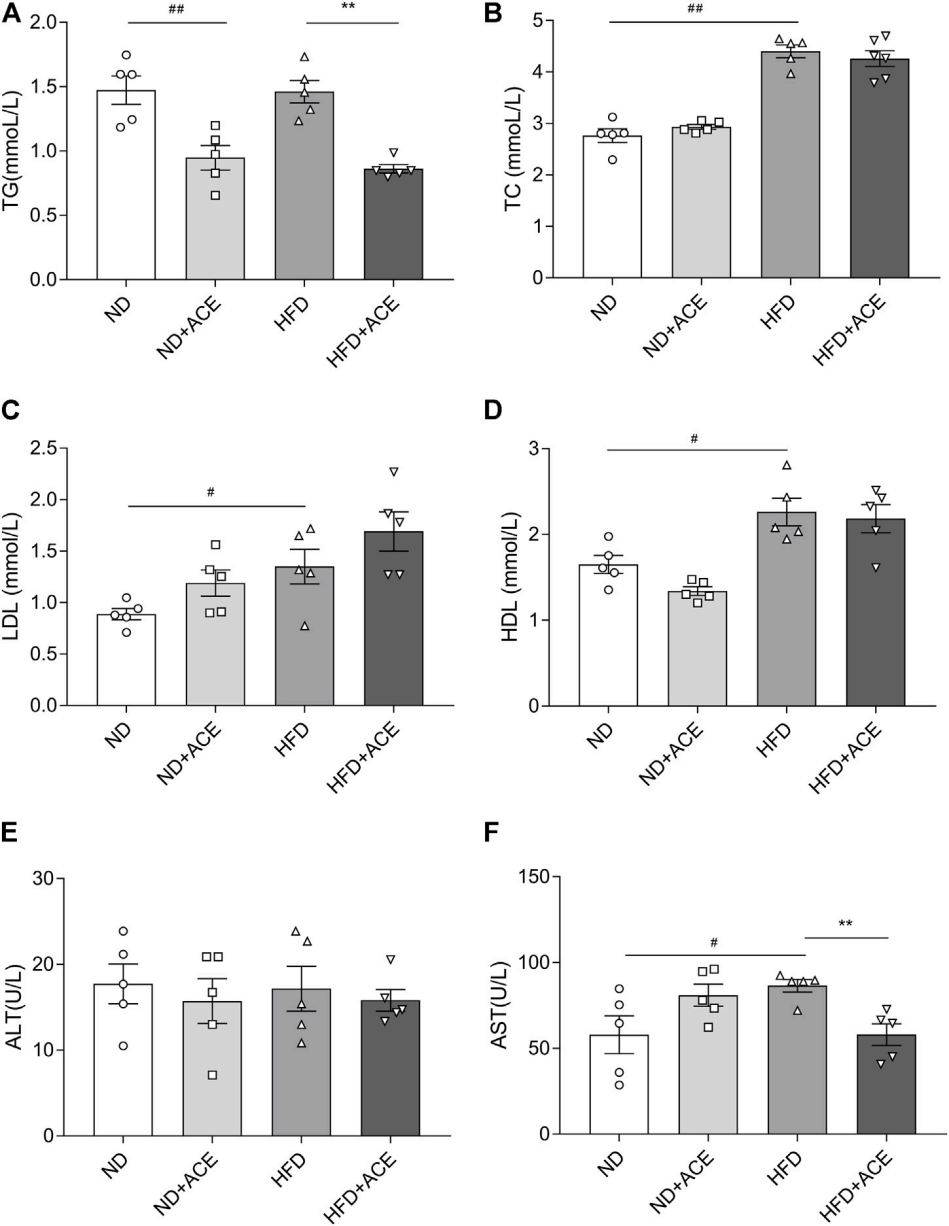
ACE ameliorated HFD-induced abnormal blood metabolic parameters. **(A)** TG content in plasma. **(B)** TC content in plasma. **(C)** LDL content in plasma. **(D)** HDL content in plasma. **(E)** ALT content in plasma. **(F)** AST content in plasma. Data were shown as means ± S.E.M (n = 5 per group). Statistical analysis was conducted by using an independent student-*t* test. ^#^denotes *p* < 0.05 when compared with the ND group, ^*##*^denotes *p* < 0.01 when compared with the ND group; *denotes *p* < 0.05 when compared with the HFD group, **denotes *p* < 0.01 when compared with the HFD group.

Lipid profile (TG, TC, HDL, LDL) and biochemical parameters for liver function evaluation were all assessed in the plasma of mice. As shown in Figure 2, HFD induced evident increases in the levels of the plasma TC, HDL, LDL, and AST as compared with the ND group (*p* < 0.05). However, no significant differences were observed in TG and ALT between these two groups. The TG level of mice in the ND + ACE group was sharply decreased in comparison to ND group, while no other difference was found between these two groups. For the HFD-fed mice, ACE administration significantly down-regulated the TG and AST levels. Collectively, these results suggest that ACE may partially improve the dyslipidemia and liver function abnormalities induced by HFD.

### ACE ameliorated high-fat diet-induced glucose intolerance and insulin resistance

**FIGURE 3:**
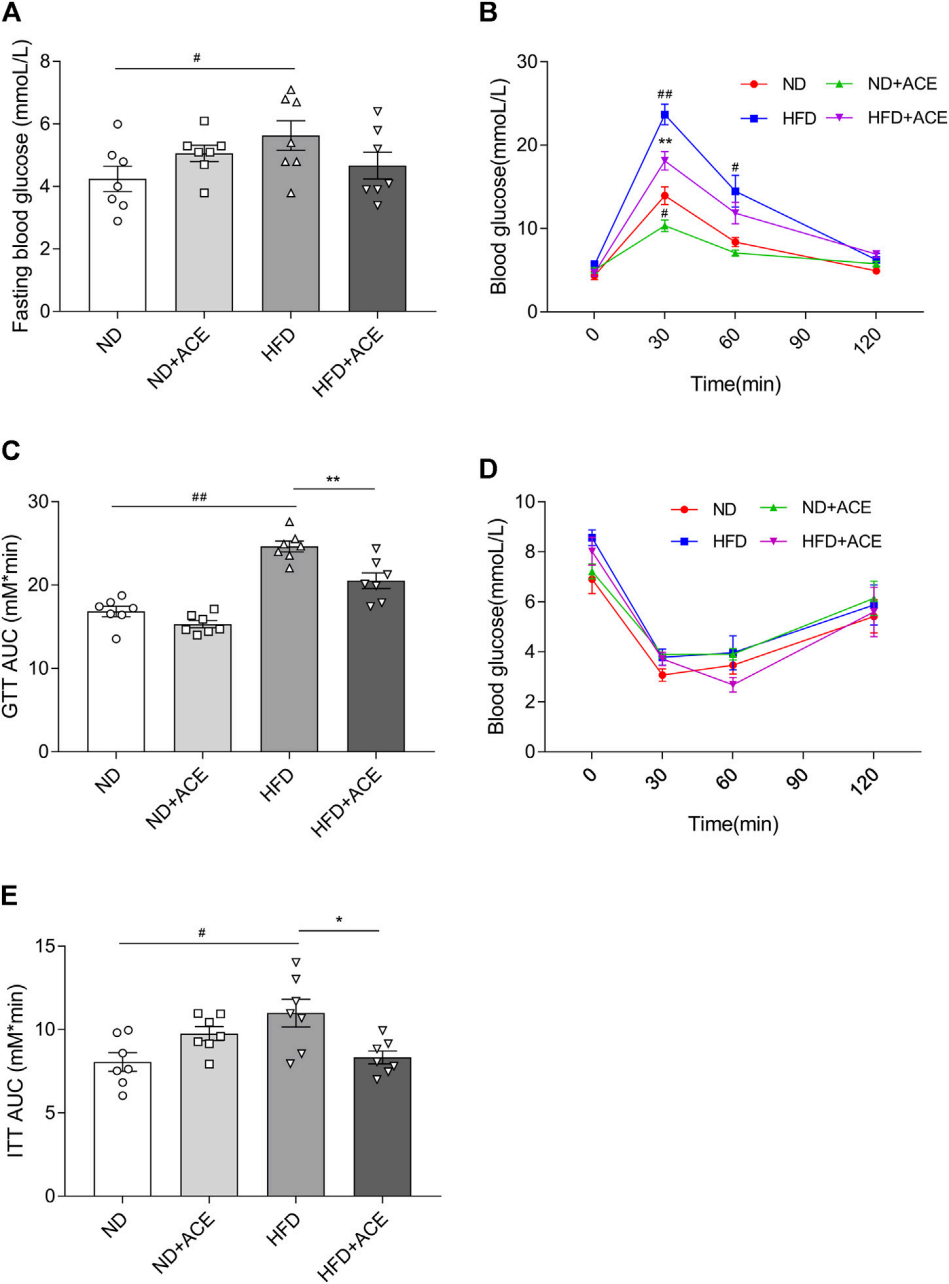
ACE ameliorated HFD-induced glucose intolerance and insulin resistance. **(A)** Fasting blood glucose levels. **(B–C)** Glucose tolerance of mice. **(D–E)** Insulin tolerance of mice. Data were shown as means ± S.E.M (n = 7 per group). Glucose tolerance test and insulin resistance test were measured at 0, 30, 60 and 120 min and statistical analysis was performed using two-way analysis of variance. Statistical analysis of the area under the curve (AUC) was conducted by using an independent student-*t* test. ^*#*^denotes *p* < 0.05 when compared with the ND group, ^*##*^denotes *p* < 0.01 when compared with the ND group; *denotes *p* < 0.05 when compared with the HFD group, **denotes *p* < 0.01 when compared with the HFD group.

Results of the glucose tolerance and insulin tolerance tests showed that HFD significantly increased the fasting blood glucose level of mice, but no difference was observed after ACE administration (*p* < 0.05; Figure 3A). Nevertheless, in comparison to the HFD group, there showed significant decreases in AUC of GTT and ITT in mice from the HFD + ACE group (*p* < 0.05; Figures 3B–E). These results together indicate ACE ameliorates glucose tolerance and insulin resistance induced by the HFD.

### ACE alleviated high-fat diet-induced liver injury

**FIGURE 4:**
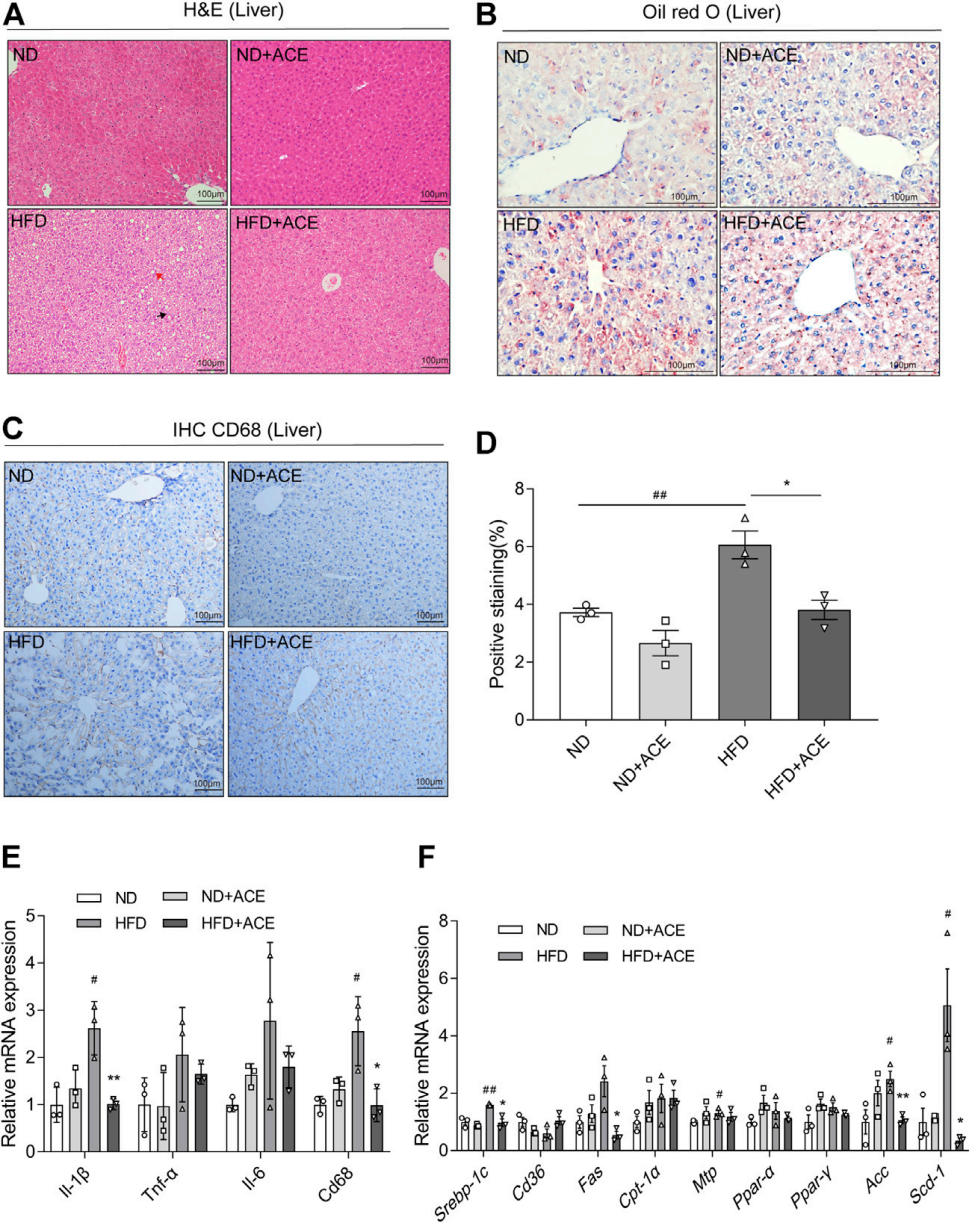
ACE alleviated HFD-induced liver injury. **(A)** Representative pictures of Hematoxylin and Eosin (H&E) staining for liver tissue. Black arrow indicates ballooning hepatocytes, red arrow indicates steatosis in liver. Pictures were shown as 20 × zoom, scale: 100 μm. **(B)** Representative pictures of Oil Red O staining for liver fat. Pictures were shown as 40 × zoom, scale: 100 μm. **(C)** Representative pictures of CD68 expression and distribution in liver tissues. Pictures were shown as 20 × zoom, scale: 100 μm. **(D)** Quantification of CD68 expression in liver tissues. **(E)** Relative expression of *Il-1β*, *Il-6*,*Tnf-α*, *Cd68* in liver. **(F)** Relative expression of *srebp-1c*, *Cd36*, *Fas*, *Cpt-1α*, *Mtp*, *Ppar-α*, *Ppar-γ*, *Acc*, *Scd-1* in liver. Data were shown as means ± S.E.M (n = 3 per group). Statistical analysis was conducted by using ANOVA or independent student-*t* test. ^#^denotes *p* < 0.05 when compared with the ND group, ^*##*^denotes *p* < 0.01 when compared with the ND group; *denotes *p* < 0.05 when compared with the HFD group, **denotes *p* < 0.01 when compared with the HFD group.

Histology analysis revealed that mice in the HFD group had more severe micro-vesicular steatosis in hepatocytes than that in the ND group (Figure 4A). Meanwhile, severe lipid droplet accumulation was observed in liver tissues of the HFD group (Figure 4B). ACE supplementation induced obvious alleviation of HFD-induced hepatic steatosis and lipid droplet accumulation.

The immunohistochemistry analysis of CD68 in liver tissues displayed that HFD upregulated CD68 expression, while ACE significantly ameliorated this effect (Figures 4C,D). Moreover, the mRNA expression levels of *Il-1β, Cd68* in the liver of HFD-fed mice were significantly increased than the mice fed with ND, while ACE administration significantly reduced their levels (*p* < 0.05; Figure 4E). To elucidate the potential mechanism of ACE-mediated reduction of hepatic lipid accumulation, the expressions of genes related to hepatic lipid metabolism were determined. Compared with the ND group, the mRNA levels of lipogenic genes *Srebp-1c*, *Mtp*, *Acc*, and *Scd-1* were significantly upregulated by HFD, whereas ACE administration downregulated the mRNA expression of *Srebp-1c*, *Fas*, *Acc*, and *Scd-1* (*p* < 0.05; Figure 4F). These findings indicate that the HFD-induced hepatic inflammation and fat metabolism abnormalities may be alleviated by ACE supplementation.

### ACE ameliorated the high-fat diet-induced adipose tissue injury

**FIGURE 5:**
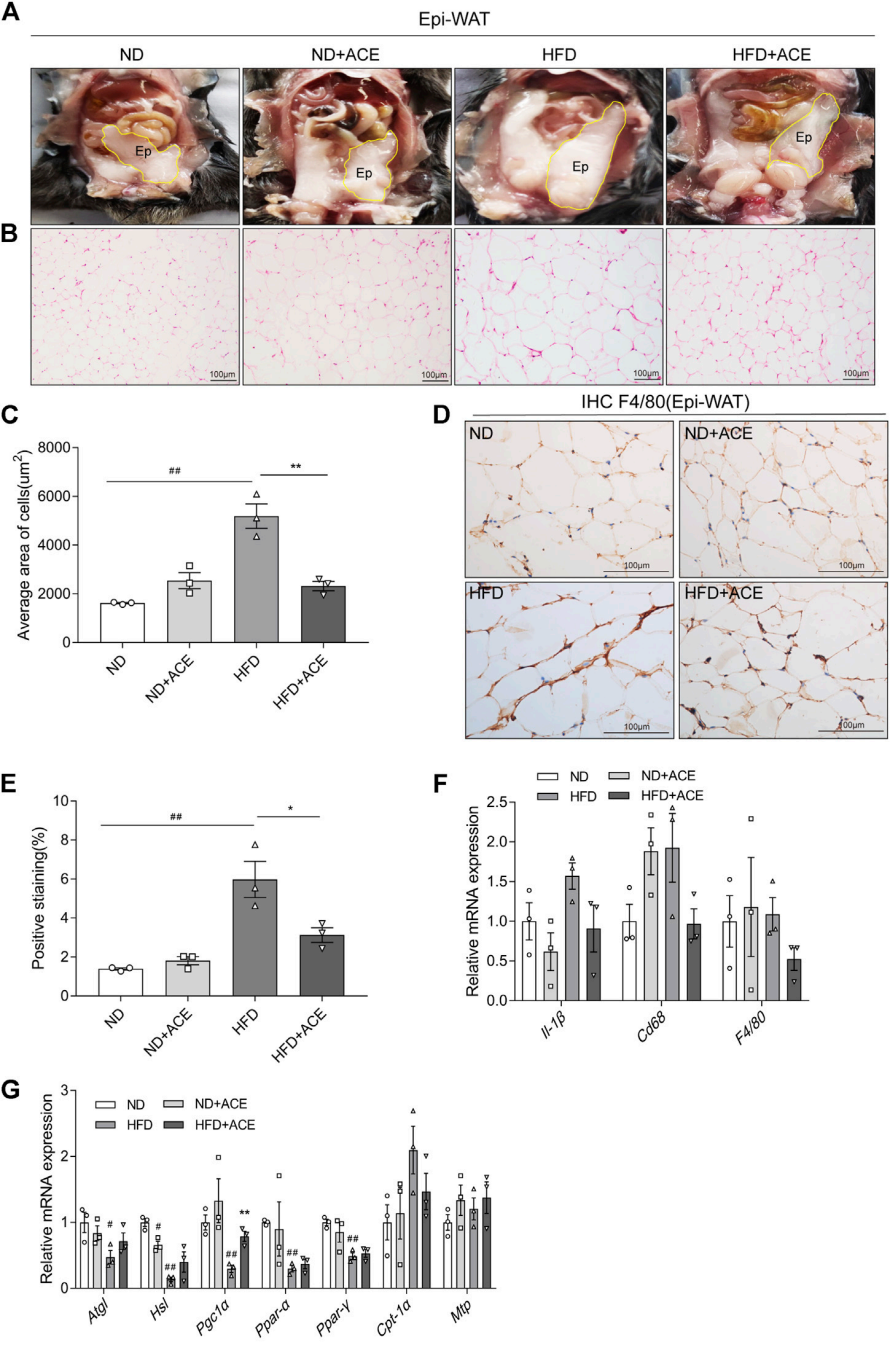
ACE ameliorated HFD-induced adipose tissue injury. **(A)** Representative pictures of dissected mouse abdomens with Epi-WAT highlighted in yellow. **(B)** Representative pictures of Hematoxylin and Eosin (H&E) staining for Epi-WAT tissue. Pictures were shown as 20 × zoom, scale: 100 μm. **(C)** The mean Adipocyte area. **(D)** Representative pictures of F4/80 expression in Epi-WAT tissues Pictures were shown as 40 × zoom, scale: 100 μm. **(E)** Quantification (% of positive stained area) of F4/80 in Epi-WAT tissues. **(F)** Relative expression of *Il-1β*, *Cd68*, *F4/80* in adipose tissue. **(G)** Relative expression of *Atgl*, *Hsl*, *Pgc1α*, *Cpt-1α*, *Mtp*, *Ppar-α*, *Ppar-γ* in adipose tissue. Data were shown as means ± S.E.M (n = 3 per group). Statistical analysis was conducted by using an independent student-*t* test. ^*#*^denotes *p* < 0.05 when compared with the ND group, ^*##*^denotes *p* < 0.01 when compared with the ND group; *denotes *p* < 0.05 when compared with the HFD group, **denotes *p* < 0.01 when compared with the HFD group.

As shown in Figure 5A, despite ACE did not induce evident changes of Epi-WAT deposit in mice fed with ND, it significantly decreased Epi-WAT deposit in the HFD-fed mice. H&E analysis revealed that Epi-WAT tissues of HFD-fed mice presented larger adipose cell sizes than those in the ND group. Notably, compared with HFD group, ACE supplementation apparently ameliorated adipocyte hypertrophy and decreased the cell size as well as the number of adipocytes in adipose tissues (*p* < 0.05; Figures 5B,C). Immunohistochemical analysis showed that HFD upregulated F4/80 expression, while ACE significantly ameliorated this effect (Figures 5D,E). Compared with the ND group, the mRNA levels of pro-inflammatory cytokines did not change in the adipose tissues of HFD-fed mice, and no significant changes were observed after ACE administration (Figure 5F). Results regarding lipid metabolism showed that HFD significantly decreased the mRNA levels of genes including *Atgl*, *Hsl*, *Pgc1α*, *Ppar-α*, and *Ppar-γ*. After treatment with ACE, the mRNA level of *Pgc1α* in adipose was significantly increased compared to the HFD group (Figure 5G). These results suggest that ACE supplementation may ameliorate the fat metabolic disturbances and relieve the inflammation in the adipose tissues of HFD-fed mice.

### ACE changed the composition of gut microbiota in high-fat diet-fed mice

**FIGURE 6:**
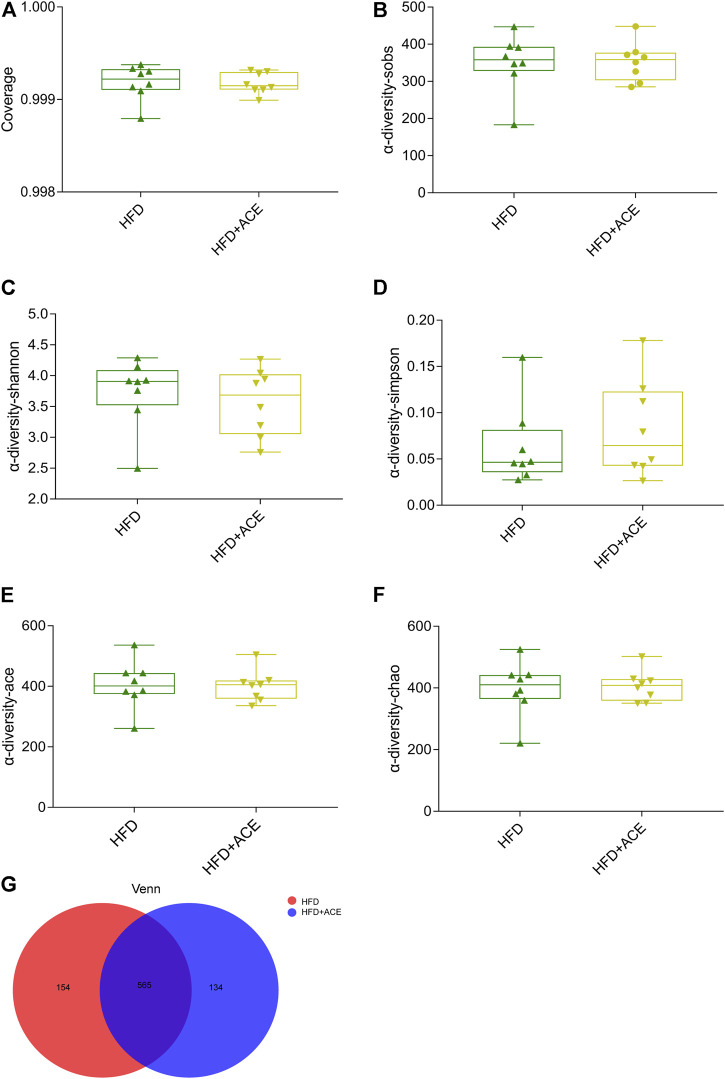
ACE did not change the richness and evenness of gut microbiota in HFD-fed mice. **(A)** The coverage indices of four groups. **(B–F)** Sobs, Shannon, Simpson, Ace, and Chao were determined to assess the *a*-diversity of gut microbiota. **(G)** Venn diagram. Data were shown as means ± S.E.M (n = 8 per group). Statistical analysis was calculated by using an independent student-*t* test.

**FIGURE 7:**
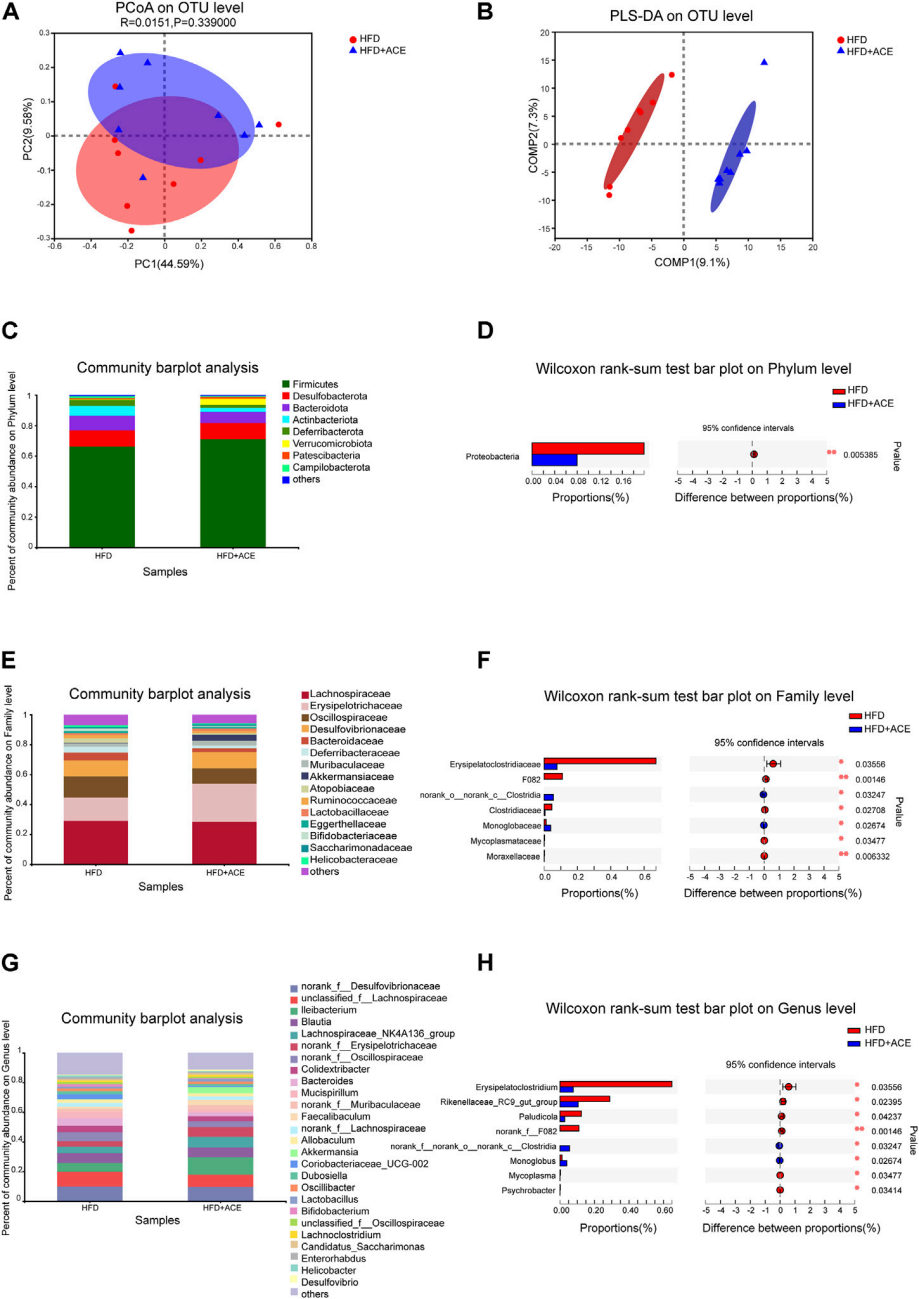
ACE changed the composition of gut microbiota in HFD-fed mice. **(A)** Principal coordinate analysis (PCoA) on OTU level. **(B)** Partial least squares discrimination analysis (PLS-DA) on OTU level. **(C)** Average relative abundance at the phylum level in each group. **(D)** The statistical analysis of microbiota at the phylum level. **(E)** Average relative abundance at the family level in each group. **(F)** The statistical analysis of microbiota at the family level. **(G)** Average relative abundance at the genus level in each group. **(H)** The statistical analysis of microbiota at the genus level. Statistical analysis was performed using Wilcoxon rank-sum test (n = 8 per group). *denotes *p* < 0.05; **denotes *p* < 0.01.

To explore the effect of ACE on HFD-induced alternation of intestinal microbial species, bacterial DNA in feces was collected and 16S rRNA gene sequencing was used to analyze the intestinal microbial composition. A total of 2,280,147 high-quality sequencing reads were obtained from 16 fecal samples (range, 96982–175117; the average number of sequences reads, 142509). Microbial community analysis was clustered into OTUs based on a 97% similarity threshold. As a result, the rarefaction curves and shannon index showed significant progressivity (Supplementary data: Supplementary Figures S1A–B), and the coverage indices of two groups suggested a near-complete sampling of the community (Figure 6A). α-diversity is an intuitive indicator for the assessment of microbial diversity, through which the level of abundance and homogeneity of the intestinal microflora were displayed. In this study, compared with the HFD group, five indices (sobs index, shannon index, simpson index, ace index, and chao index) were not significant changed after ACE supplementation (Figures 6B–F). These data together imply that exposure to ACE does not affect α-diversity of gut microbiota. As shown in the Venn’s diagram (Figure 6G), the HFD group and HFD + ACE group shared the compositional overlap of 565 core microbiota. These overlapping phylotypes contributed to 78.6% (565/719) and 80.8% (565/699) of HFD group and HFD + ACE group, respectively. Principal coordinate analysis (PCoA) was applied to analysis the dissimilarities in microbial composition on OTU level. As shown in Figure 7A, samples in the HFD + ACE group were primarily concentrated on the up side, whereas the samples in the HFD group presented mainly on the below side. Partial least squares discrimination analysis (PLS-DA) further distinguished samples of the HFD + ACE group from those in the HFD group, suggesting there exist significant changes in the gut microbial community composition between these two groups (Figure 7B).

To further explore the effects of ACE on intestinal microbial species, the gut microbiota was analyzed by 16S rRNA pyrosequencing at the phylum, class, order, family and genus levels. At phylum level, ACE administration significantly reduced the abundance of *Proteobacteria* in comparison to the HFD-treated group (*p* < 0.05; Figures 7C,D). However, no change in the *Firmicutes/Bacteroidetes* was observed after ACE administration (Supplementary data: Supplementary Figure S1C). At the class level, compared with the HFD group, HFD + ACE significantly reduced the abundance of *Gammaproteobacteria* (*p* < 0.05; Supplementary data: Supplementary Figures S1D–E). In addition, at the order level, significant differences were observed in *Clostridiales, Monoglobales, Mycoplasmatales, Pseudomonadales,* and *Micrococcales* between the two groups (*p* < 0.05; Supplementary data: Supplementary Figures S1F–G). At the family level, samples in the HFD + ACE group had significant changes in the abundance of *Erysipelatoclostridiaceae* *, F082, norank_o_norank_c_Clostridia,* *Clostridiaceae,* *Monoglobaceae, Mycoplasmataceae*, and *Moraxellaceae* compared to those of the HFD group (Figures 7E,F). While at the genus level, ACE administration significant changed the abundance of *Erysipelatoclostridium, Rikenellaceae* *_RC9_gut_group, Paludicola, norank_f_F082, norank_f_ norank_o_norank_c_Clostridia, Monoglobus, Mycoplasma,* and *Psychrobacter* when compared with the HFD group (Figures 7G,H).

To clarify the changes of gut microbiota at different levels, we compared the gut microbiota of HFD group and HFD + ACE group using the linear discriminant analysis (LDA) effect size (LEfSe) method. A cladogram representative of the structure of gut microbiota and their predominant bacteria were shown in Supplementary data: Supplementary Figure S1H. As shown in Supplementary data: Supplementary Figure S1I, Linear discriminant analysis (LDA) coupled with effect size (LEfSe) established 24 bacterial clades showing statistically significant and biologically consistent differences (LDA score ≥2.0) from phylum to genus level. These results further verify that ACE administration can reshape the composition of intestinal microbial that were altered by the HFD.

## Discussion

Nowadays, obesity has become a global public health crisis nationally and internationally (James, 2018). A lot of efforts have been made to explore strategies to prevent and manage obesity. Improving eating habits, increasing physical activity, going for bariatric surgery or taking anti-obesity medication are the main choices (Lin and Li, 2021). For medication use, phentermine, orlistat, liraglutide 3.0 are commonly used for weight loss, among which phentermine is only United States Food and Drug Administration (FDA)-approved for short-term use (3 months). Numerous adverse effects such as nausea, dry mouth, difficulty sleeping, constipation and irritability may occur from taking these medications (Tchang et al., 2021). Thus, these side effects hampered the use of these medications. Accordingly, recent studies have concentrated on the identification of candidate drugs, particularly focused on the natural herbs. *Asparagus cochinchinensis* is a traditional Chinese medicine which has therapeutic properties including antioxidant, anti-aging, anti-tumor and blood sugar reduction without inducing obvious side effects (Zhao et al., 2012; Lei et al., 2016; Lee et al., 2018). In the present study, the HFD-induced obese mouse model with or without ACE treatment were established, and the results revealed that ACE administration reduced body weight gain caused by the HFD. Furthermore, the improved glucose tolerance, insulin resistance, lipid metabolism and inflammatory response indicate ACE might contribute to weight loss based on these changes. In addition, the high-throughput sequencing of 16S rRNA gene analysis suggested that ACE could reshape the gut microbiota, which might be another explanation for its anti-obesity effects. To our knowledge, this is the first study to suggest the anti-obesity potential of ACE.

Consistent with previous findings, *Asparagus cochinchinensis* administration alleviated TG levels as well as glucose tolerance and insulin resistance induced by HFD, suggesting an anti-obesogenic and antidiabetic potential of *Asparagus cochinchinensis* (Martel et al., 2017). The liver is a major metabolic organ in the body, it plays an important role in lipid metabolism such as endogenous fat production, *in vivo* fat transport and storage (Hodson et al., 2020). HFD can increase the infiltration of hepatic macrophages and locally produce inflammatory chemokines and cytokines, which are related to a spectrum of liver abnormalities like hepatic steatosis (Andrade et al., 2014; Wu et al., 2020). Herein, we observed that elevated *Il-1β*, *Cd68* in HFD animal model were significantly down-regulated in the liver after ACE administration. These findings were in line with previous report which revealed ACE can effectively reduce inflammatory damage in skin of mice (Lee et al., 2009). Increasing evidence has showed that genes related to fatty acid synthesis and metabolism in liver will be changed by HFD, and anti-obesity candidates may exert an anti-obesity effect by regulating the expression of these genes (Ipsen et al., 2018). The results of the present study revealed that ACE treatment decreased the HFD-induced up-regulation of *Srebp-1c*, *Fas*, *Acc*, and *Scd-1* in liver of mice, which were important regulators of lipid metabolism in liver tissue (Higuchi et al., 2008; Chen et al., 2019). The decreased expression of these genes may at least partially explain the improvement on fatty degeneration observed after ACE administration.

In addition to the liver tissue, the adipose tissue is essential for controlling systemic homeostasis (Choe et al., 2016). In the process of excessive nutrients intake, fat cells enlarged and proliferated, accompanied by the increased immune cells and elevated inflammatory cytokine levels (Britton and Fox, 2011; Choe et al., 2016). In this study, ACE administration significantly reduced the expression of macrophage markers and genes related to fat metabolism, which were up-regulated in mice fed with HFD. According to these results, we speculated that ACE could reduce fat deposition and inhibit inflammation in HFD-fed mice. This is consistent with the results of a previous study, where researchers observed an inhibitory effect of ACE against airway inflammation in an asthma mouse model (Sung et al., 2017).

Gut microbiota has emerged as a possible endogenous factor which impacts the development of obesity (Gerard, 2016). Indeed, gut microbiota modulation has become a new paradigm for tackling obesity (Tremaroli and Backhed, 2012). Interestingly, multiple Chinese herbal medicine has been shown to improve obesity and the obesity-correlated metabolic diseases by beneficially regulating the gut microbiota (Payab et al., 2018). In this study, ACE administration appeared to reshape the intestinal microbial composition of the HFD-fed mice. Normal intestinal microflora is mainly composed of *Firmicutes* and *Bacteroidetes*, and both of them are important in regulating host carbohydrate, lipid and bile acid metabolism (Tremaroli and Backhed, 2012). The abundance of *Firmicutes/Bacteroidetes* are related to the susceptibility of disease, but there is still controversy about whether they are associated with obesity (Riva et al., 2017). Turnbaugh et al. reported lower *Bacteroides* levels in obese subjects compared with lean subjects, but no significant differences were observed in the abundance of *Firmicutes* (Turnbaugh et al., 2009). However, Ley et al. examined the microbiome of obese human subjects before and after weight loss, and found that weight loss was associated with the decrease of *Firmicutes* and the increase of *Bacteroidetes* (Ley et al., 2006). In the present study, we found no significant difference in *Firmicutes/Bacteroidetes* in the HFD-fed mice after ACE administration, which was consistent with the results of Fernandes et al. (Fernandes et al., 2014). These inconsistencies may be due to a combination of large interpersonal variation and methodological differences among studies. We also found that the abundance of *Proteobacteria* in HFD-fed mice decreased significantly after ACE administration. A healthy individual contains only a minor proportion of *Proteobacteria*. In the cases like diet-induced obesity, metabolic disorders and inflammation, a bloom of *Proteobacteria* was found (Shin et al., 2015). We speculate the anti-obesity effect of ACE may be related to the decreased *Proteobacteria*. This hypothesis is also supported by a previous study, where the researchers observed the obesogenic potential of *Proteobacteria* in a mono association study in germ-free mice, indicating a correlation between metabolic disorder and the expansion of *Proteobacteria* (Fei and Zhao, 2013).

We found ACE administration significantly reduced the abundance of *Clostridiaceae* in the HFD-fed mice, which was consistent with the results of Tolnai et al., indicating *Clostridiaceae* exerted a negative effect on weight loss (Tolnai et al., 2021). However, there is still controversy about the effect of *Clostridiaceae* on obesity. Louis et al. revealed that *Clostridiaceae* produced butyrate, while the content of butyrate in the intestinal tract was suggested to be negatively correlated with the formation of obesity (Louis and Flint, 2017). Apart from the alternation of *Clostridiaceae*, ACE administration significantly reduced the abundance of *Pseudomonadales* in intestinal flora of obese mice. Members of the *Pseudomonadales* order include opportunistic Gram-negative pathogens, which could trigger an innate immune response in the murine host. Nevertheless, the mechanisms underlying the impact of *Pseudomonadales* on obesity still deserve further investigation (Damron et al., 2016). The significantly reduced *Erysipelotrichaceae* in the HFD-fed mice after ACE administration was another important finding in this study. *Erysipelotrichaceae* can enhance the energy extraction from diet and shape the lipidemic profiles of hosts (Etxeberria et al., 2015). Therefore, the decrease of this bacterium observed after ACE administration may be responsible for weight loss. At genus level, we observed significantly increased *Monoglobus* in the HFD-fed mice after ACE administration. *Monoglobus* is positively correlated with anti-inflammatory factors, indicating the up-regulation of *Monoglobus* may be a reason for the reduced inflammation level caused by ACE. (Festa et al., 2001; Wang et al., 2022).

In this study, LC-MS was used to identify the monomeric compounds in ACE. The results showed that 76 monomer compounds mainly belonging to steroidal saponins, flavonoids, lipids were observed. Unfortunately, due to the limitation of LC-MS, the exact contents of these ingredients were not quantified. According to recent reports, 19 amino acids, polysaccharides, and >20 multi-functional compounds were observed in *Asparagus cochinchinensis* root (Zhang et al., 2004; Lee et al., 2017). Steroidal saponins is considered to be the main active ingredient, which was also observed in our study (Jalsrai et al., 2016). As revealed in recent reports, steroidal saponins can regulate intestinal microbiota and ameliorate obesity-induced pathology. Therefore, steroidal saponins from ACE may be involved in improving the obesity-related indicators and gut microbiota composition. Inulin-type fructan named ACNP (*Asparagus cochinchinensis* neutral polysaccharide) obtained from *Asparagus cochinchinensis* root, was revealed to be beneficial to human intestinal microbiota, which was also observed in our study (Sun et al., 2020). This evidence may provide some clues for the beneficial effects observed in obesity-related indicators and gut microbiota composition after ACE administration. Due to the complex composition of ACE, further studies are still needed to quantitative analysis of the components and determine specific effects of individual bioactive components on obesity phenotypes and microbial communities. Notably, ACE also contains toxic ingredients (Zeiner and Juranović Cindrić, 2017). The extract obtained by traditional methods may inevitably contain these substances. Improved technology may be promising to minimize the unwanted substances (Li et al., 2020; Zhang et al., 2021). The dose selection of Chinese herbal medicine is a key factor to ensure clinical efficacy and safety, thus the dose of Chinese herbal medicine must be adjusted to adapt to different disease conditions in a clinic (Zha et al., 2015). To the best of our knowledge, this study investigated the anti-obesity effect of ACE for the first time. Thus, a therapeutic relevance dose of 450 mg/kg/d was selected based on previous reports (Sung et al., 2016; Sung et al., 2017). Nevertheless, this is a relatively high dose because a range of 100–200 mg/kg of extracts were assumed to be the upper limit for pharmacologically meaningful levels in the *in vivo* studies (Heinrich et al., 2020). Therefore, finding a low therapeutically effective dose is an important future work. Additionally, the effect of ACE was evaluated only at one dose in this study, which may limit the comprehensive understanding its effects. Thus, it is still necessary to explore the effects of ACE with series doses in future work.

## Conclusion

In summary, the results herein demonstrated the anti-obesity effect of ACE on HFD-fed mice. ACE administration effectively reduced the elevated body weight, visceral adipose tissue weight, and blood lipid levels induced by HFD. This beneficial effect may attribute to the improved lipid metabolism, suppressed inflammatory response and modulated gut microbiota induced by ACE. Collectively, our findings suggest the effect of ACE in the prevention and treatment of obesity, indicating the potential of ACE to be used in improving health.

## Data Availability

The datasets presented in this study can be found in online repositories. The names of the repository/repositories and accession number(s) can be found in the article/Supplementary Material.

## Abbreviations

ACE, aqueous extract from Asparagus cochinchinensis root; ALT, Alanine aminotransferase; AST, Aspartate aminotransferase; Acc, Acetyl-CoA carboxylase; Atgl, Adipose triglyceride lipase; CD68, Cluster of differentiation 68; Cd36, Cluster of differentiation 36; Cpt-1α, Carnitine palmitoyltransferase 1 alpha; Epi-WAT, epididymal white adipose tissue; Fas, Fatty acid synthase; F4/80, Adhesion G protein-coupled receptor E1; HFD, High-fat diet; HFD + ACE, oral aqueous extract from Asparagus cochinchinensis root group under high-fat diet; HDL, High-density lipoprotein cholesterol; Hsl, Hormone-sensitive lipase; Il-1β, Interleukin 1bata; Il-6, Interleukin 6; LDL, Low-density lipoprotein cholesterol; Mtp, Microsomal triglyceride transfer protein; ND, Normal diet; ND + ACE, oral aqueous extract from Asparagus cochinchinensis root group under normal diet; Srebp-1c, Sterol regulatory element-binding protein 1c; Scd-1, Stearoyl-CoA desaturase-1; Ppar-α;, Peroxisome proliferator-activated receptor alpha; Ppar-γ;, Peroxisome proliferator-activated receptor gamma; Pgc1α, Peroxisome proliferator-activated receptorγcoactivator-1; Per-WAT, perirenal white adipose tissue; TG, Triglyceride; TC, Total cholesterol; Tnf-α, Tumor necrosis factor alpha; β-actin, beta-actin.
