# Supplementary material for: Asparagus cochinchinensis alleviates disturbances of lipid metabolism and gut microbiota in high-fat diet-induced obesity mice
Source: Front Pharmacol. 2022 Oct 12;13:1015005. doi: 10.3389/fphar.2022.1015005 (PMC9616603; doi:10.3389/fphar.2022.1015005)
Supplement: Supplementary file 2 [file Table1.docx]

**Table S3.** Primer sequences used in qRT-PCR

| **Gene** | **Forward primer (5’-3’)** | **Reverse primer (5’-3’)** |
| --- | --- | --- |
| β-actin | CATCCGTAAAGACCTCTATGCCAAC | ATGGAGCCACCGATCCACA |
| Pgc1α | TTGATGTGAATGACTTGGATA | GTTGTACTGGTTGGATATGA |
| Hsl | TGGAACATCACTGAGATTG | AGGTGAGATGGTAACTGT |
| Atgl | CGTGGCTGTCTACTAAAG | GCTAAAGTGGGATATGATGA |
| Il-1β | TGCCACCTTTTGACAGTGAT | TGTCCTCATCCTGGAAGGTC |
| Il-6 | AGTTGCCTTCTTGGGACTGA | CCACGATTTCCCAGAGAAC |
| Tnf-α | GGCAGGTCTACTTTGGAGTCATTGC | ACATTCGAGGCTCCAGTGAATTCGG |
| Cd68 | TGTCTGATCTTGCTAGGACCG | GAGAGTAACGGCCTTTTTGTGA |
| F4/80 | GAGTGGAATGTCAAGATGTTA | CAGTGGAAGAAGAGAAGC |
| FAS | CCCGGAGTCGCTTGAGTATATT | GGACCGAGTAATGCCATTCAG |
| Scd-1 | CAGTGCCGCGCATCTCT | CCCGGGATTGAATCTTCTTG |
| Acc | GTCCCGGCCACATAACTGAT | CGCTCAGGTCACCAAAAAGAAT |
| Cd36 | CTTACACATACAGAGTTCGTTATC | TCCAACAGACAGTGAAGG |
| Mtp | CCGCTGTGCTTGCAGAAGA | TTTGACACTATTTTTCCTGCTATGGT |
| Ppar-α | CGGCAGTGCCCTGAACA | TGGTACCCTGAGGCCTTGTC |
| Ppar-γ | CACAATGCCATCAGGTTTGG | GCTGGTCGATATCACTGGAGATC |
| Cpt-1α | GAACCCCAACATCCCCAAAC | TCCTGGCATTCTCCTGGAAT |
| Srebp-1c | CATGCCATGGGCAAGTACAC | TGTTGCCATGGAGATAGCATCT |
