# Supplementary figures and images for: Asparagus cochinchinensis alleviates disturbances of lipid metabolism and gut microbiota in high-fat diet-induced obesity mice
Source: Front Pharmacol. 2022 Oct 12;13:1015005. doi: 10.3389/fphar.2022.1015005 (PMC9616603; doi:10.3389/fphar.2022.1015005)

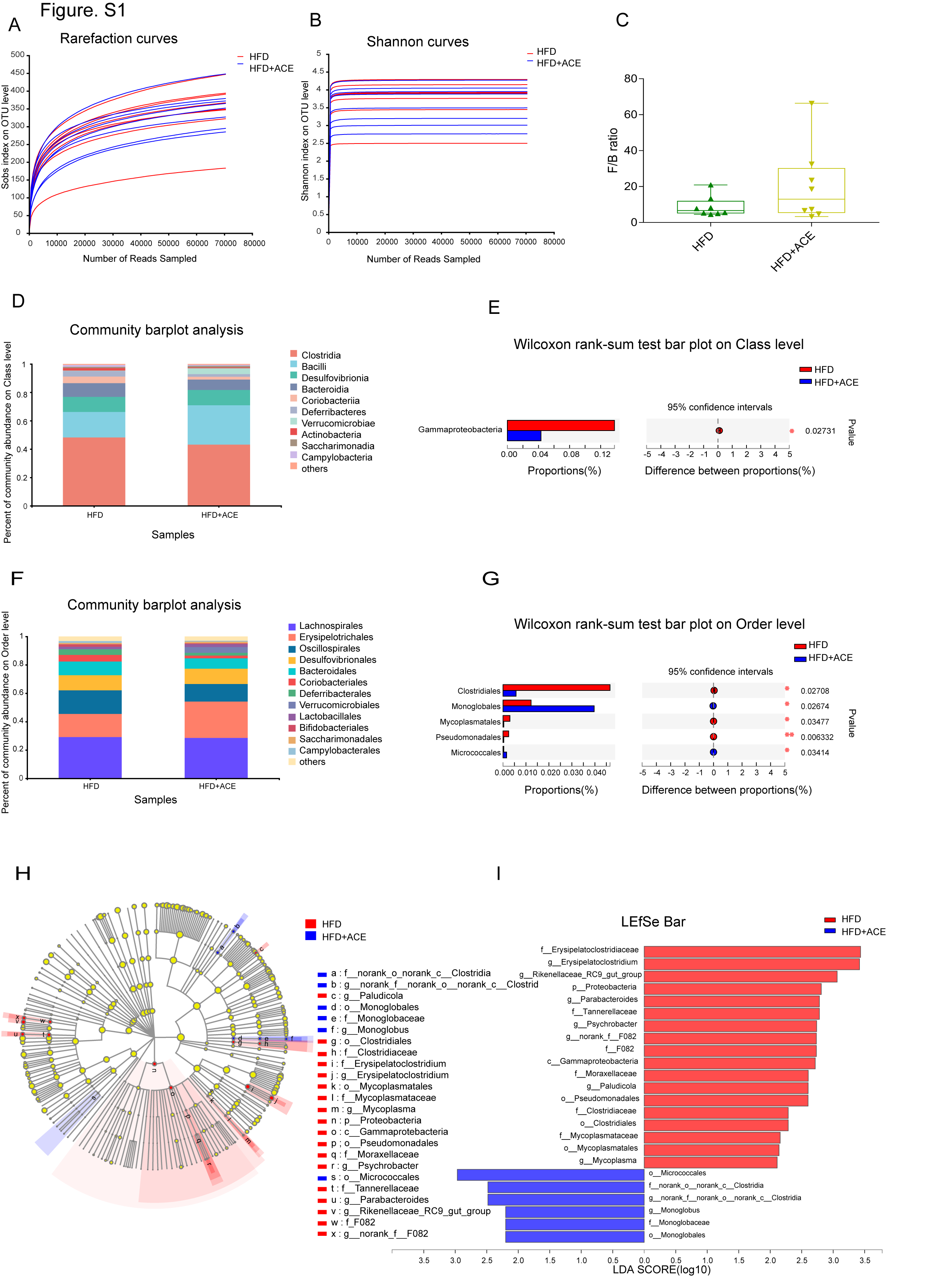

Supplement: Supplementary file 3 [file Image1.TIF]
