# Supplementary material for: Asparagus cochinchinensis alleviates disturbances of lipid metabolism and gut microbiota in high-fat diet-induced obesity mice
Source: Front Pharmacol. 2022 Oct 12;13:1015005. doi: 10.3389/fphar.2022.1015005 (PMC9616603; doi:10.3389/fphar.2022.1015005)
Supplement: Supplementary file 4 [file DataSheet1.PDF]

Supplementary Material

1 Supplementary Tables and Figures

1.1 Supplementary Tables

Supplementary Table S1. The mobile phase gradient of LC-MS.

Supplementary Table S2. Analysis of the extract of ACE by LC-MS.

Supplementary Table S3. Primer sequences used in qRT-PCR.

Supplementary Figure S1. The composition of gut microbiota in mice from different groups. **(A)** The rarefaction curves of two groups. **(B)** The shannon curves of two groups. **(C)** *Firmicutes/Bacteroidetes* ratio of two groups. **(D)** Average relative abundance at the class level in each group. **(E)** The statistical analysis of microbiota at the class level. **(F)** Average relative abundance at the order level in each group. **(G)** The statistical analysis of microbiota at the order level. **(H)** The bacterial clades shown with the taxonomic cladogram. Biomarker taxa are highlighted by colored circles and shaded areas. Each circle's diameter reflects the abundance of that taxa in the community. **(I)** Bacterial clades between two groups analyzed by Linear discriminant analysis (LDA) coupled with effect size (LEfSe). Statistical analysis of *Firmicutes/Bacteroidetes* ratio was conducted by using an independent student-t test, and data were shown as means ± S.E.M (n = 8 per group). Statistical analysis was conducted at the Class and Orde taxonomical level by using Wilcoxon rank-sum test, separately (n = 8 per group). $*P < 0.05$; $**P < 0.01$; $***P < 0.001$.
