# Supplementary material for: Asparagus cochinchinensis alleviates disturbances of lipid metabolism and gut microbiota in high-fat diet-induced obesity mice
Source: Front Pharmacol. 2022 Oct 12;13:1015005. doi: 10.3389/fphar.2022.1015005 (PMC9616603; doi:10.3389/fphar.2022.1015005)
Supplement: Supplementary file 5 [file Table3.DOCX]

**Table S3.** Primer sequences used in qRT-PCR

| **Gene** | **Forward primer (5’-3’)** | **Reverse primer (5’-3’)** |
| --- | --- | --- |
| β-actin | CATCCGTAAAGACCTCTATGCCAAC | ATGGAGCCACCGATCCACA |
| PGC1α | TTGATGTGAATGACTTGGATA | GTTGTACTGGTTGGATATGA |
| HSL | TGGAACATCACTGAGATTG | AGGTGAGATGGTAACTGT |
| ATGL | CGTGGCTGTCTACTAAAG | GCTAAAGTGGGATATGATGA |
| IL-1β | TGCCACCTTTTGACAGTGAT | TGTCCTCATCCTGGAAGGTC |
| IL-6 | AGTTGCCTTCTTGGGACTGA | CCACGATTTCCCAGAGAAC |
| TNF-α | GGCAGGTCTACTTTGGAGTCATTGC | ACATTCGAGGCTCCAGTGAATTCGG |
| CD68 | TGTCTGATCTTGCTAGGACCG | GAGAGTAACGGCCTTTTTGTGA |
| F4/80 | GAGTGGAATGTCAAGATGTTA | CAGTGGAAGAAGAGAAGC |
| FAS | CCCGGAGTCGCTTGAGTATATT | GGACCGAGTAATGCCATTCAG |
| SCD-1 | CAGTGCCGCGCATCTCT | CCCGGGATTGAATCTTCTTG |
| ACC | GTCCCGGCCACATAACTGAT | CGCTCAGGTCACCAAAAAGAAT |
| CD36 | CTTACACATACAGAGTTCGTTATC | TCCAACAGACAGTGAAGG |
| MTP | CCGCTGTGCTTGCAGAAGA | TTTGACACTATTTTTCCTGCTATGGT |
| PPAR-α | CGGCAGTGCCCTGAACA | TGGTACCCTGAGGCCTTGTC |
| PPAR-γ | CACAATGCCATCAGGTTTGG | GCTGGTCGATATCACTGGAGATC |
| CPT-1α | GAACCCCAACATCCCCAAAC | TCCTGGCATTCTCCTGGAAT |
| SREBP-1c | CATGCCATGGGCAAGTACAC | TGTTGCCATGGAGATAGCATCT |
